# Distribution analysis of tetracycline resistance genes in *Escherichia coli* isolated from floor surface and effluent of pig slaughterhouses in Banten Province, Indonesia

**DOI:** 10.14202/vetworld.2023.509-517

**Published:** 2023-03-21

**Authors:** Debby Fadhilah Pazra, Hadri Latif, Chaerul Basri, I. Wayan Teguh Wibawan, Puji Rahayu

**Affiliations:** 1 Animal Biomedical Science Study Program, School of Veterinary Medicine and Biomedical Sciences (SVMBS), IPB University, Bogor, Indonesia; 2 Animal Health Study Program, Bogor Agricultural Development Polytechnic, Bogor, Indonesia; 3 Department of Animal Diseases and Veterinary Public Health, School of Veterinary Medicine and Biomedical Sciences (SVMBS), IPB University, Bogor, Indonesia; 4 Quality Control Laboratory and Certification of Animal Products, Bogor, Indonesia

**Keywords:** effluent, *Escherichia coli*, pig slaughterhouse, *tet* gene, tetracycline resistance

## Abstract

**Background and Aim:**

Slaughterhouses and their effluents could serve as a “hotspot” for the occurrence and distribution of antibiotic-resistant bacteria in the environment. This study aimed to understand the distribution of tetracycline resistance genes in *Escherichia coli* isolated from the floor surface and effluent samples of pig slaughterhouses in Banten Province, Indonesia.

**Materials and Methods:**

Ten samples, each from floor surface swabs and effluents, were collected from 10 pig slaughterhouses in Banten Province. *Escherichia coli* strains were isolated and identified by referring to the protocol of the *Global Tricycle Surveillance* extended-spectrum beta-lactamase *E. coli* from the WHO (2021). Quantitative real-time polymerase chain reaction (qPCR) was used to detect the *tet* genes.

**Results:**

The *tetA*, *tetB*, *tetC*, *tetM*, *tetO*, and *tetX* genes were distributed in the isolates from the floor surface samples, and the *tetA*, *tetC*, *tetE*, *tetM*, *tetO*, and *tetX* genes were distributed in the isolates from the effluent samples. The *tetO* gene (60%) was the most dominant gene in the isolates from floor surface samples, while the *tetA* gene was the dominant one in the isolates from the effluent samples (50%). The *tetA* + *tetO* gene combination was the dominant pattern (15%) in the *E. coli* isolates.

**Conclusion:**

The high prevalence and diversity of the *tet* genes in floor surface and effluent samples from pig slaughterhouses in Banten Province indicated that the transmission of the *tet* genes had occurred from pigs to the environment; thus, this situation should be considered a serious threat to public health.

## Introduction

The emergence and spread of antibiotic resistance due to the overuse of antibiotics in the farming industry pose a serious global threat to public health. Some studies have reported that both resistant pathogenic and commensal bacteria can be transmitted through the consumption of animal-based food products and environmental contamination [1]. Deaths due to infections caused by drug-resistant bacteria are estimated to increase yearly and reach 700,000–10,00,000 persons annually by 2050. Furthermore, global economic loss due to antibiotic resistance is likely to reach 100 trillion USD by 2050 [1]. Infection with multidrug-resistant bacteria renders the treatment ineffective; this eventually increases material loss, diminishes the quality of life, causes death of the infected individuals, and reduces the success rate of health improvement programs [2].

The crossover use of medically important antibiotics in livestock is considered one of the reasons for the emergence of antibiotic-resistant bacteria and their transmission to humans and the environment. Tetracycline is a commonly used antibiotic in humans because of its broad spectrum of activity, easy availability, and low cost. According to Jurnalis *et al*. [3], tetracycline is the most common antibiotic class available in public healthcare centers. Kallau *et al*. [4] and Detha *et al*. [5] reported that tetracycline is the common antibiotic used to treat pigs in Indonesia. Some studies have shown that bacterial species resist tetracycline through their *tet* genes [6, 7].

Effluents from slaughterhouses are known to be “hotspots” for the development and spread of antibiotic-resistant bacteria, resulting in the transfer of antibiotic resistance genes (ARGs) to other bacterial species [8]. The animal slaughtering process produces abundant liquid waste (1000 L/1000 kg live animal mass), and this liquid waste is potentially contaminated with antibiotic-resistant bacteria [8, 9]. The incomplete removal of antibiotic-resistant bacteria from wastewater treatment plants leads to the transportation of these bacteria along with the liquid waste to larger water bodies and terrestrial areas, resulting in environmental pollution, especially of aquatic systems, and eventually reaching humans through the contaminated food chain [10–12].

*Escherichia coli*, Gram-negative enteric commensal bacteria, are commonly found in humans and animals. *Escherichia coli* is one of the 12 enlisted critical priority pathogens. It can transmit ARGs to other bacteria, regardless of the species, horizontally through mobile genetic elements (MGEs) or vertically through replication [13, 14]. *Escherichia coli* is an indicator organism mostly used to determine the level of microbial contamination of water [15] and as an indicator to monitor antimicrobial resistance [13, 14].

Banten Province in Indonesia has several pig slaughterhouses centered in Tangerang City. The untreated effluent from pig slaughterhouses has a high potential to pollute the environment and ecosystem and poses a serious threat to public health. The contaminated floor where slaughtering is performed also acts as a source of contamination of the pork by drug-resistant bacteria. At present, limited data are available regarding the presence of antibiotic-resistant *E. coli* and ARGs, particularly tetracycline resistance genes, in pig slaughterhouses.

This study aimed to understand the distribution of tetracycline resistance genes in *E. coli* isolated from floor surface and effluent samples of pig slaughterhouses in Banten Province, Indonesia. The study findings can be used as a base to develop appropriate strategies to control antibiotic resistance.

## Materials and Methods

### Ethical approval

Ethical approval was not required for this study. The samples were collected in accordance with the guidelines of the standards for sample collection procedure (SNI 6989.59-2008 and ISO 19458:2006) [16, 17].

### Study period and location

The study was conducted from July to October 2022. The isolation and identification of *E. coli* from the samples were performed at the microbiology laboratory of SKHB IPB. Tetracycline resistance genes were detected by quantitative real-time polymerase chain reaction (qPCR) at the Quality Control Laboratory and Certification of Animal Products, Ministry of Agriculture, Republic of Indonesia.

### Sample collection

All of the pig slaughterhouses in Banten Province were taken as samples (as many as 10 pig slaughterhouses). Ten samples each of floor surface swab and effluent were collected. The effluent was sampled in accordance with the standards SNI 6989.59-2008 for the wastewater sampling method [16] and ISO 19458:2006 for sample collection for microbiological analysis of water quality [17]. Samples were collected aseptically and transported to the laboratory while being preserved at 4°C. The effluent at pig slaughterhouses was collected at two different time points: During the slaughtering process and after completion of the process; 500 mL sample was collected at each time point (total sample volume: 1 L).

### Isolation and identification of *E. coli*

*Escherichia coli* was isolated and identified in accordance with the protocol of the *Global Tricycle Surveillance* extended-spectrum beta-lactamase *E. coli* from WHO [18]. All samples were serially diluted up to 10^-5^ dilution in duplicate by using sterile phosphate-buffered saline (PBS; pH 7.4) in a ratio of 1:9. Next, 0.1 mL of the sample from each dilution was added to a petri dish containing tryptone bile X-glucuronide (TBX) agar (Merck, Germany) and plated on the surface using the spread plate method. Bluish-green colonies on the TBX agar plate were suspected to be of *E. coli*. Petri dishes with a colony count of ≤100 colony-forming unit (CFU)/mL were used for subsequent analysis. Five bluish-green colonies from each TBX agar plate were inoculated into MacConkey agar (MCA; Oxoid, UK) plates. The suspected *E. coli* colonies on MCA plates appeared as morphologically flat, dry, pink, and nonmucoid colonies with a surrounding darker pink area of precipitated bile salts. The suspected *E. coli* colony was cultured on a tryptic soy agar medium (Oxoid) and then cultured on sulfide indole motility medium (Oxoid) to perform the indole test to confirm *E. coli*. The formation of a cherry red ring in the indole test was considered to be a positive confirmation of the *E. coli* isolate. *Escherichia coli* ATCC 25922 was used as a positive control.

### DNA extraction

DNA from the *E. coli* isolate was extracted using the Mericon DNA Bacteria Kit (Qiagen, Germany) in accordance with the manufacturer’s protocol. The pure *E. coli* isolate was transferred using an inoculating loop from the culture medium into a microtube containing 1 mL of sterile PBS to achieve a turbidity of 0.5 McFarland standard or more (depending on the availability of the isolate). The suspension was centrifuged at 13,000× *g* for 5 min. The supernatant was discarded using a pipette, and 200 μL of sterile PBS was added to the bacterial pellet; the mixture was homogenized using a vortex mixer. Subsequently, the suspension was centrifuged at 13.000× *g* for 5 min. The bacterial pellet was washed several times to obtain a colorless suspension. Next, 200 μL of Fast Lysis Buffer was added, and the mixture was placed in a ThermoMixer (Eppendorf, Germany) for heating at 100°C at the rotation speed of 122× *g* for 10 min. The suspension was then incubated at room temperature (TM) for 2 min. The resulting suspension was centrifuged at 13,000× *g* for 5 min. Subsequently, 100 μL DNA-containing supernatant was transferred into a 2 mL microtube and incubated at –20°C or –80°C until further analysis.

### Quality control of the extracted DNA

DNA concentration and purity were tested using a nanodrop spectrophotometer. The DNA purity ratio assessed by nanodrop was considered appropriate when it matched the set value of 1.8–2.0 (A_260_/A_280_). The amount of DNA concentration needed for the qPCR test was >36 ng/μL.

### Detection of tetracycline resistance genes

Tetracycline resistance genes were detected using the qPCR SYBR Green method with primers of the target genes (Table-1 [19–21]). A real-time PCR thermal cycler (Rotor-Gene Q, Germany) was used for this method. The reagents were added to microtubes according to the required experimental design as follows: 12 μL of SYBR select master mix, 2 μL of 10 μM reverse primer, 2 μL of 10 μM forward primer, 3.5 μL of nuclease-free water, and 5 μL of DNA sample. The total reaction volume was 25 μL. Each microtube was placed on a PCR plate cooler to keep the reagent at low TM. In the qPCR SYBR Green method, melting was performed using Q-Rex software (Qiagen). The *tetA*, *tetM*, *tetO*, and *tetX* genes were amplified with the procedure proposed by Li *et al*. [22] using a two-step qPCR program with the following protocol: 3 min of initial heating at 95°C followed by 40 cycles of denaturation for 10 s at 95°C, 60 s of annealing at the TM adjusted for the primers listed in Table-1, and extension at 72°C for 1 min. The *tetB*, *tetC*, and *tetE* genes were amplified by referring to the procedure of Jia *et al*. [23] as follows: Initial heating at 94°C for 5 min, followed by 40 cycles of denaturation for 60 s at 94°C, 30 s of annealing at the TM adjusted for the primers listed in Table-1 [19–21], and extension at 72°C for 90 s. The specificity of the amplified product was analyzed using the melting curve (95°C for 10 s, 65°C–95°C with 0.5°C increment every 0.05 s).

**Table-1:** Details of the primers used to detect tetracycline resistance genes.

Gene	Primer	Primer sequence (5'–3')	Temperature annealing (°C)	Reference
*tetA*	*tetA*-F	GCTACATCCTGCTTGCCTTC	57	[19]
	*tetA*-R	CATAGATCGCCGTGAAGAGG		
*tetB*	*tetB*-F	TTG GTT AGG GGC AAG TTT TG	56	[19]
	*tetB*-R	GTA ATG GGC CAA TAA CAC CG		
*tetC*	*tetC*-F	CTTGAGAGCCTTCAACCCAG	55	[19]
	*tetC*-R	ATGGTCGTCATCTACCTGCC		
*tetE*	*tetE*-F	AAACCACATCCTCCATACGC	57	[19]
	*tetE*-F	AAATAGGCCACAACCGTCAG		
*tetO*	*tetO*-F	ACGGARAGTTTATTGTATACC	57	[20]
	*tetO*-R	TGGCGTATCTATAATGTTGAC		
*tetM*	*tetM*-F	ACAGAAAGCTTATTATATAAC	52	[20]
	*tetM*-R	TGGCGTGTCTATGATGTTCAC		
*tetX*	*tetX*-F	AGCCTTACCAATGGGTGTAAA	57	[21]
	*tetX*-R	TTCTTACCTTGGACATCCCG		

The result was considered positive if the cycle threshold (CT) value was <40 with the amplification curve and a single melt peak was formed with melting TM equal to or the tolerance value of TM <2°C. The result was considered negative/undetectable if the CT value was >40 without the amplification curve. The result was considered indeterminate if the CT value was between >36 and <40.

### Statistical analysis

The experimental data were presented as tables and figures and analyzed using a descriptive approach.

## Results

### Isolation and identification of *E. coli*

All the tested samples (ten floor surface samples and ten effluent samples) were positive for *E. coli*. Table-2 shows the complete results of the isolation and identification of *E. coli*.

**Table-2:** Results of *E. coli* isolation and identification.

Sample type	Amount isolate culture	Testing stages			Positive *E. coli* (%)

		TBX media culture (%)	MCA media culture (%)	Indole test (%)	
Floor surface	10	10 (100)	10 (100)	10 (100)	100%
Effluent	10	10 (100)	10 (100)	10 (100)	100%

*E. coli*=*Escherichia coli*, TBX=Tryptone bile X-glucuronide, MCA=MacConkey agar

### Distribution of tetracycline resistance genes

**Figure-1:**
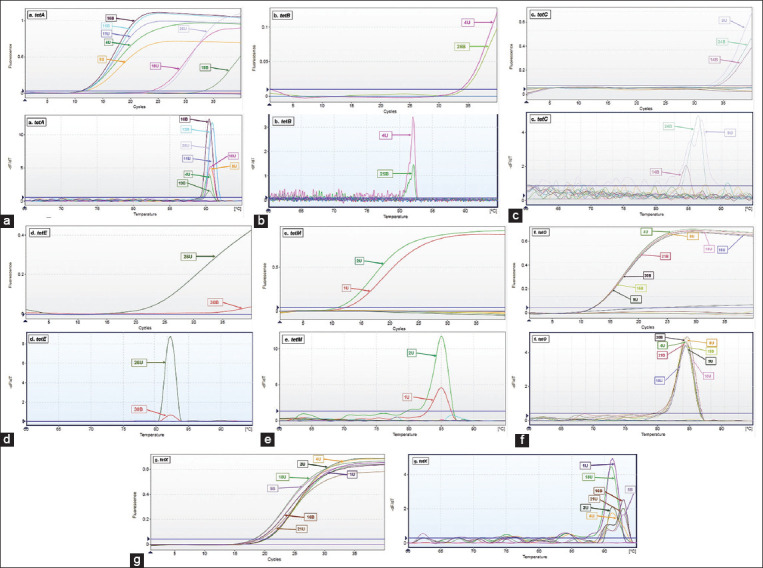
Test results showing the detection of *tet* genes in floor surface and effluent samples by quantitative real-time polymerase chain reaction. (a) Amplification curve and melting curve of *tetA*; (b) amplification curve and melting curve of *tetB*; (c) amplification curve and melting curve of *tetC*; (d) amplification curve and melting curve of *tetE*; (e) amplification curve and melting curve of *tetM*; (f) amplification curve and melting curve of *tetO*; (g) amplification curve and melting curve of *tetX*.

The *tetA*, *tetB*, *tetC*, *tetM*, *tetO*, and *tetX* genes were present in the *E. coli* isolates from floor surface samples, while the *tetA*, *tetC*, *tetE*, *tetM*, *tetO*, and *tetX* genes were detected in the *E. coli* isolates from effluent samples (Table-3). Figure-1 shows the amplification curve and melting curve from the test result of tetracycline resistance genes determined using qPCR.

**Table-3:** Cycle threshold and melt peak values of the *tet* genes detected in the floor surface and effluent samples using qPCR.

Floor surface sample code	*tet* genes	CT value	*Melt* *peak* (°C)	*Effluent* sample code	*tet* genes	CT value	*Melt* *peak* (°C)
1U	*tetM*	12.27	85.0	2U	*tetM*	10.90	85.0
	*tetX*	20.15	91.3		*tetX*	19.99	91.3
4U	*tetA*	11.16	90.4	5B	*tetX*	20.29	93.0
	*tetB*	29.99	82.1	8U	*tetA*	11.89	90.6
	*tetO*	11.88	84.3		*tetO*	12.09	84.3
	*tetX*	20.32	91.3	11U	*tetA*	11.29	90.9
9U	*tetC*	31.78	87.9	14B	*tetC*	33.94	85.8
	*tetO*	12.01	84.3	16B	*tetA*	11.10	90.5
10U	*tetA*	23.73	90.6		*tetX*	20.15	93.0
	*tetO*	12.24	84.8	19B	*tetA*	33.22	90.5
15B	*tetA*	11.73	90.9	26U	*tetA*	24.76	90.8
	*tetO*	12.03	84.5		*tetE*	15.78	83.5
18U	*tetO*	11.93	84.8	30B	*tetE*	29.42	84.1
	*tetX*	19.99	91.3		*tetO*	11.96	85.0
21B	*tetO*	11.96	84.5				
	*tetX*	20.29	93.0				
24B	*tetC*	33.25	87.0				
25B	*tetB*	30.48	82.1				

qPCR=Quantitative real-time polymerase chain reaction, CT=Cycle threshold

**Figure-2:**
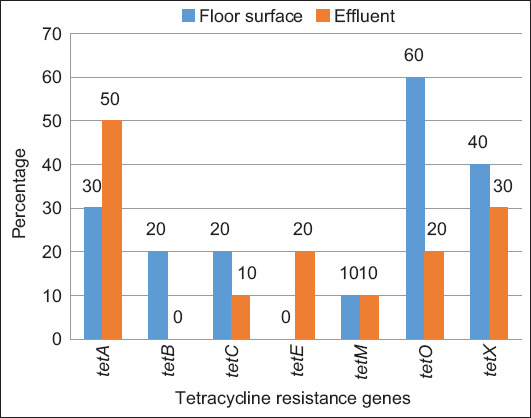
Prevalence percentage of tetracycline resistance genes in floor surface and effluent samples from pig slaughterhouses.

**Figure-3:**
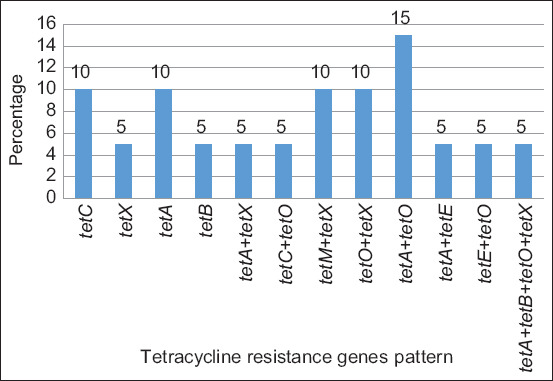
Pattern of tetracycline resistance genes in samples from pig slaughterhouses.

The *tetO* gene was the most dominant one and detected in 60% of floor surface samples, followed by *tetX* (40%), *tetA* (30%), *tetB* and *tetC* (20%), and *tetM* (10%), while the *tetE* gene was not detected (0%). In the effluent samples, the *tetA* gene was the most dominant one and detected in 50% of samples, followed by *tetX* (30%), *tetE* and *tetO* (20%), and *tetC* and *tetM* (10%), while the *tetB* gene was not detected (0%) (Figure-2). Twelve *tet* gene patterns were distributed between the samples from the pig slaughterhouses; the dominant pattern was *tetA* + *tetO* (15%), followed by *tetA*, *tetC*, *tetM* + *tetX*, and *tetO* + *tetX* (10%) and *tetB*, *tetX*, *tetA* + *tetX*, *tetC* + *tetO*, *tetA* + *tetE*, *tetE* + *tetO*, and *tetA* + *tetB* + *tetO* + *tetX* (5%) (Figure-3).

## Discussion

### Isolation and identification of *E. coli*

The floor surface and effluent samples from the ten pig slaughterhouses showed a high prevalence (100%) of *E. coli*. Researchers from several countries have also reported a high prevalence of *E. coli* in pig slaughterhouses. Savin *et al*. [24] reported a high prevalence (85.1%) of *E. coli* in wastewater treatment plants of pig slaughterhouses in Germany. A high prevalence of *E. coli* was also observed in the waste of pig slaughterhouses in Portugal [25] and in the effluent of slaughterhouses in Addis Ababa, Ethiopia [26]. Blaak *et al*. [27] showed that the percentage of *E. coli* in several slaughterhouses varied depending on different factors, such as the condition of the sampling area, fecal contamination level, sampling period, effluent flow rate, and disposal conditions.

In this study, the high prevalence of *E. coli* (100%) in floor surface and effluent samples from pig slaughterhouses was caused by a high rate of fecal contamination from the intestines of the slaughtered animals and poor health condition of the slaughtered animals [28]. The large amount of *E. coli* detected in the samples of floor surfaces and effluent of pig slaughterhouses might be caused by the abundance of blood traces (high protein medium), which could be an ideal source of nutrition for *E. coli* to grow and reproduce [29]. The presence of *E. coli* also suggests the possibility of the presence of other pathogens critical to public health safety [30]. Poor hygiene and sanitation conditions at pig slaughterhouses led to the spread of *E. coli* from animals, humans, the environment, and ecosystems through contaminated waste. In Banten Province, most of the effluent from pig slaughterhouses is disposed to Cisandane River, without pretreatment of the waste; this practice poses a serious risk to people who use the river water for their daily needs. A study on Cisandane River by Purwati *et al*. [31] showed a high level of *E. coli* contamination between 2 × 10^2^ and 3 × 10^5^ CFU/100 mL.

### Distribution of tetracycline resistance genes

The qPCR SYBR Green method used in the present study showed positive results if the CT value was <40, an amplification curve was formed, and there was a single melt peak with equal TM value or the tolerance of TM value was <2°C. This concept was based on the studies of Ririe *et al*. [32] and Dortmans *et al*. [33] who reported that melting curve analysis could differentiate amplification products with the same length but have different GC/AT ratios with TM values <2°C. Melting point is generally acceptable in the range of 0.5–2°C. The narrow range (0.5°C–2°C) of melting point usually indicates the purity of the testing materials. The melting peak values of all the tested *tet* genes were as follows: *tetA*, 90.53°C; *tetB*, 82.0°C; *tetC*, 87.0°C; *tetE*, 84.11°C; *tetM*, 85.04°C; *tetO*, 84.45°C; and *tetX*, 91.28°C.

Melting curve analysis using high-resolution DNA melting analysis (HRM) generates a DNA melt curve profile that enables DNA characterization based on sequence length, guanine and cytosine as the main components, and complementarity of the DNA sequence. This method is very sensitive for classification based on variations in nucleic acid sequences or for the detection of changes in a single-nucleotide base that would form a melting curve with different patterns; thus, this method is useful to detect mutations or identify other genetic variants [34]. The difference in the melting curve pattern in the HRM method is a reflection of various DNA sequences of the target genes in each isolate [35]. This method can detect different nucleic acid compositions without sequencing, and thus, it is relatively cheaper than other conventional techniques [36, 37].

The present study tested seven types of *tet* genes that induce resistance to tetracycline through different mechanisms: Efflux pump (*tetA*, *tetB*, *tetC*, and *tetE*), ribosomal protection (*tetM* and *tetO*), and enzymatic inactivation (*tetX*). *Tet* genes are responsible for the emergence of resistance to tetracycline antibiotics in *E. coli*. Among the seven *tet* genes tested, almost all of them, except the *tetE* gene, were detected in the floor surface samples, and the *tetB* gene was detected in the effluent samples. The *tetO* gene (60%) was the dominant gene in the floor surface samples, whereas *tetA* was the dominant one in the effluent samples (50%). Savin *et al*. [38] demonstrated the presence of the *tetA*, *tetB*, and *tetM* genes in the effluent of pig slaughterhouses in Germany, but the *tetX* gene was not detected. Similar results have been reported in Portugal [25], wherein the *tetA*, *tetB*, *tetM*, and *tetK* genes were detected in the waste of a pig farm, and the *tetA*, *tetB*, *tetK*, *tetL*, *tetM*, *tetO*, and *tetA(P)* genes were detected in the effluent of slaughterhouses; the *tetX* gene was not found in both pig farm waste and slaughterhouse effluent in Portugal.

The *tetA*, *tetB*, *tetC*, *tetD*, *tetE*, and *tetG* genes are the dominant genes that induce tetracycline resistance in *E. coli* through the efflux pump mechanism that actively pumps out tetracycline to the periplasm during proton (H^+^) exchange by active transport, resulting in resistance due to inadequate concentration of the antibiotic, which subsequently prevents the binding of tRNA to the A-site of the ribosome 30S subunit and inhibits protein synthesis [39]. The *tetA* gene is the dominant efflux pump gene, followed *tetC*, and the least dominant ones are *tetB* and *tetE*. This finding agrees with the results of most other studies which stated that the *tetA* gene was indeed the most dominant one to be present in Gram-negative bacteria [40] and was often detected in pigs [41] or other animals [42, 43], meats, processed meat products [44], and various environmental samples [25, 45]. The *tetA* gene is present in the conjugation plasmid, from where it can easily transmit resistance genes to other bacterial species throughout conjugative horizontal gene transfer. The genes *tetC* and *tetE* are also present in the bacterial plasmid, while the *tetB* gene is present in the transposon and integrative and conjugative elements (ICE) [46]. The *tetE* gene is often associated with the nonconjugative plasmid [21] because of its limited transmission. In the present study, the percentage occurrence of the *tetE* gene was much lower than that of the other efflux pump genes, and it was even undetectable in the floor surface samples.

The *tetO* and *tetM* genes were the *tet* genes responsible for the ribosomal protection resistance mechanism, which is known as ribosomal protection proteins or RPPs that protect the ribosome by disrupting the main binding site of tetracycline [47, 48]. The *tetO* gene was the dominant gene in the floor surface samples (60%), but it showed a low occurrence (20%) in the effluent samples. Similarly, the *tetM* gene showed a low percentage of occurrences in both floor surface and effluent samples (10%). Amador *et al*. [25] detected *tetO* in *E. coli* isolated from slaughterhouse effluent, even though it had a low percentage of occurrences (12.5%), while the gene *tetM*, in contrast, was detected in a quite high percentage (43.8%).

The *tetO* gene is mostly associated with the conjugation plasmid in *Campylobacter* spp., and an inter-isolate transfer of *tetO* in *Campylobacter jejuni* has also been reported [49]. The *tetO* gene is also associated with the hot plasmid that was mediated within *Enterococcus* and *Streptococcus* spp. [50]. A recent study revealed that the *tetO* gene was integrated into transposons carrying the macrolide-resistant efflux genes *mefA* and *msrD*. This transposon can be transferred conjugatively to different strains of *Streptococcus pyogenes* and the unrelated *Enterococcus faecalis* [51, 52]. The *tetO* gene is rarely found in *E. coli*; however, in the present study, a high prevalence of this gene was detected, particularly in floor surface samples. This most likely occurred because the conjugative transposon carrying the *tetO* gene had a wider transmission among other unrelated bacteria, such as *E. coli*. As reported by Roberts [48], *tetO* found on the conjugative transposon could possibly cause much wider transmission of this gene among various unrelated bacteria in the future. Antibiotic resistance genes associated with a conjugative transposon can be easily transmitted to other bacteria, even those that are unrelated, as compared to ARGs associated with a nonconjugative plasmid [53].

The *tetX* genes responsible for the enzymatic inactivation resistance mechanism can modify the enzymatic activity, resulting in the inactivation of tetracycline. The *tetX* gene encodes a cytoplasmic protein that chemically transmutes tetracycline using oxygen and NADPH through the addition of a –OH cluster on C-11 of the tetracycline molecule [47]. At present, *tetX* has been identified in *E. coli*, although the enzymatic inactivation resistance mechanism has not been deeply observed [54]. Some studies have detected *tetX* in the effluent of pig slaughterhouses [55], manure made of pig feces [56], well water in close proximity to a pig farm [57], and river water [58].

The *tet* genes have been discovered on various MGEs, such as transposons, plasmids, integrons, and ICEs [59–61]. The abundance of MGEs carrying the *tet* genes has significantly contributed to the transmission of tetracycline-resistant bacteria to a wider environment through gene transfer from one bacterial species to another. Plasmids have contributed in transmitting multidrug resistance genes not only among bacteria of the same species but also among bacteria of unrelated species [62]. Integrons are often found on the plasmid and/or transposon that increase the transmission rate of ARGs. Class-1 integron is known to distribute ARGs among Gram-negative and Gram-positive bacteria [63]. Moredo *et al*. [64] reported that approximately 17.5% of *E. coli* isolates from pig slaughterhouses carried integrons as the transmitter of resistant genes.

In the present study, most *E. coli* isolates carried 2 *tet* genes, while some isolates had 4 *tet genes*. The dominant pattern among the combination of the *tet* genes was *tetA* + *tetO* (15%) (Figure-3). The high rate of prevalence and the variety of the *tet* genes detected in either floor surface samples or effluent samples from the 10 pig slaughterhouses in Banten Province indicated the occurrence of pig-to-environment transmission due to the overuse and uncontrolled application of tetracycline antibiotics in pig farms. Kallau *et al*. [4] showed that antibiotics are mostly used in pig farms for medication (55.21%), disease prevention (42.71%), and enhanced production (2.08%). Antibiotics of tetracycline class (oxytetracycline and tetracycline) are frequently used in pig farms [4, 65, 66].

The effluent of slaughterhouses could function as a “hotspot” for the growth and reproduction of different bacterial species as well as for the transmission and exchange of ARGs at a high level among different bacterial cells, thereby resulting in various combinations of such genes. The *E. coli* strain carrying multiple tetracycline resistance genes might increase the probability of forming a new combination or pattern of the *tet* genes, if this condition persists. The occurrence of new *tet* gene combinations is a very serious concern with unpredictable consequences on human health and environment [67].

The presence of antibiotic-resistant *E. coli* in pigs might cause contamination of the pork [66], processed meat products [68], and pig slaughterhouse environment and its waste [38], in addition to having the potential to transmit to a wider area and ecosystem [57, 58, 69]. Marshall and Levy [70] and Sanders *et al*. [11] showed that ARGs could be distributed to agricultural lands by direct and indirect contact through food, water, and animal feces.

## Conclusion

The *tetO* gene (60%) was the most dominantly distributed gene in floor surface samples, while *tetA* (50%) was the dominant one in the effluent samples. The dominant pattern in the combination of the *tet* genes in the *E. coli* isolates was *tetA* + *tetO* (15%). The high prevalence and diversity of the *tet* genes in floor surface and effluent samples from pig slaughterhouses in Banten Province indicated that the transmission of the *tet* genes had occurred from pigs to the environment. In addition, poor waste management could be a source of transmission of *E. coli* that carries ARGs to the environment and may pose a serious threat to public health.

## Authors’ Contributions

DFP: Coordinated the research, performed sample and data collection, conducted sample testing, performed data analysis, and wrote the manuscript. HL and IWTW: Involved in coordinating the research, data interpretation, and manuscript preparation and review. CB and PR: Involved in coordinating the research, data analysis, and manuscript preparation. All authors have read, reviewed, and approved the final manuscript.
